# Evolution of the *Cytomegalovirus* RL11 gene family in Old World monkeys and Great Apes

**DOI:** 10.1093/ve/veae066

**Published:** 2024-08-24

**Authors:** Ulad Litvin, Eddie C Y Wang, Richard J Stanton, Ceri A Fielding, Joseph Hughes

**Affiliations:** MRC-University of Glasgow Centre for Virus Research, Sir Michael Stoker Building, 464 Bearsden Road, Glasgow G61 1QH, United Kingdom; Division of Infection and Immunity, Cardiff University School of Medicine, UHW Main Building, Heath Park, Cardiff CF14 4XN, United Kingdom; Division of Infection and Immunity, Cardiff University School of Medicine, UHW Main Building, Heath Park, Cardiff CF14 4XN, United Kingdom; Division of Infection and Immunity, Cardiff University School of Medicine, UHW Main Building, Heath Park, Cardiff CF14 4XN, United Kingdom; MRC-University of Glasgow Centre for Virus Research, Sir Michael Stoker Building, 464 Bearsden Road, Glasgow G61 1QH, United Kingdom

**Keywords:** cytomegaloviruses, RL11 gene family, gene duplication, phylogenetics, host–virus evolution

## Abstract

*Cytomegalovirus* (CMV) is a genus of herpesviruses, members of which share a long history of coevolution with their primate hosts including New World monkeys, Old World monkeys (OWMs), and Great Apes (GAs). These viruses are ubiquitous within their host populations and establish lifelong infection in most individuals. Although asymptomatic in healthy individuals, infection poses a significant risk to individuals with a weakened or underdeveloped immune system. The genome of human CMV is the largest among human-infecting viruses and comprises at least 15 separate gene families, which may have arisen by gene duplication. Within human CMV, the RL11 gene family is the largest. RL11 genes are nonessential *in vitro* but have immune evasion roles that are likely critical to persistence *in vivo*. These genes demonstrate an extreme level of inter-species and intra-strain sequence diversity, which makes it challenging to deduce the evolutionary relationships within this gene family. Understanding the evolutionary relationships of these genes, especially accurate ortholog identification, is essential for reconstructing ancestral genomes, deciphering gene repertoire and order, and enabling reliable functional analyses across the CMV species, thereby offering insights into evolutionary processes, genetic diversity, and the functional significance of genes. In this work, we combined *in silico* genome screening with sequence-based and structure-guided phylogenetic analysis to reconstruct the evolutionary history of the RL11 gene family. We confirmed that RL11 genes are unique to OWM and GA CMVs, showing that this gene family was formed by multiple early duplication events and later lineage-specific losses. We identified four main clades of RL11 genes and showed that their expansions were mainly lineage specific and happened independently in CMVs of GAs, African OWMs, and Asian OWMs. We also identified groups of orthologous genes across the CMV tree, showing that some human CMV–specific RL11 genes emerged before the divergence of human and chimpanzee CMVs but were subsequently lost in the latter. The extensive and dynamic species–specific evolution of this gene family suggests that their functions target elements of host immunity that have similarly coevolved during speciation.

## Introduction

The cytomegaloviruses (CMVs; genus *Cytomegalovirus*, subfamily *Betaherpesvirinae*, family *Orthoherpesviridae*, and order *Herpesvirales*; Lefkowitz et al. 2018) are a group of herpesviruses that infect only primate hosts. Based on the similar topologies of *Cytomegalovirus* and primate phylogenetic trees and limited cross-species transmissions, CMVs are thought to have cospeciated with their hosts and now are restricted to them (McGeoch et al. 2006, Brito et al. 2021). However, some CMVs did switch hosts in the past several million years (Murthy et al. 2019, Brito et al. 2021). Currently, at least 11 primate species are known to be infected with species-specific CMVs (Cagliani et al. 2020), of which human CMV (HCMV, *Human betaherpesvirus 5*) is the best studied. Like other members of the *Orthoherpesviridae* family, primary infection with HCMV leads to the establishment of a lifelong latent infection, despite the induction of robust humoral and cellular immunity. Prior infection and the presence of latent virus are insufficient to prevent superinfection; thus, individuals can carry multiple strains simultaneously. Primary infection, reactivation, and reinfection are usually asymptomatic in healthy individuals (De la Hoz et al. 2002). However, HCMV poses a significant risk to people with compromised (transplant recipients undergoing immunosuppressive treatment and individuals with HIV coinfection) or immature (infants) immune systems leading to high viral loads and end-organ disease (Griffiths and Reeves 2021). According to seroprevalence studies, between 78% and 88% of the adult population worldwide is infected with HCMV; however, the percentage varies with geographical region and age group (Zuhair et al. 2019).

HCMV is an enveloped DNA virus. It has a linear double-stranded DNA genome of ∼235 kilobase pairs, one of the largest among viruses infecting animals. The genome is composed of two regions flanked by pairs of inverted repeats: a long unique region (U_L_) and a short unique region (U_S_). It carries ∼170 canonical protein–coding genes, most of which can be grouped into 15 gene families (Davison et al. 2003). The RL11 gene family is the largest and the most diverse gene family in the HCMV genome. It encompasses 14 genes (RL5A, RL6, RL11—RL13, UL1, and UL4–UL11) located at the end of the U_L_ next to the left terminal repeat. RL11 genes encode type 1 membrane glycoproteins with comparable domain architecture (Davison et al. 2003). Most of these proteins carry a signal peptide (SP), an extracellular region similar to the immunoglobulin variable domain (IgV-like domain) known as RL11D, and a transmembrane domain (TMD). However, certain regions can be missing from some proteins, e.g. RL5A and RL6 genes encode proteins without an SP and TMD, UL4 lacks a TMD and is secreted (Vlachava et al. 2023), while UL5 encodes only a TMD. Orthologous genes belonging to the RL11 family show the highest level of divergence between different HCMV genotypes of any genes in the genome (Sekulin et al. 2007). It has been reported that the RL11 gene family might have originated as the result of the primate CD229/SLAMF3/LY9 gene being co-opted into the CMV genome (Engel et al. 2011).

All members of the RL11 gene family are dispensable for HCMV reproduction *in vitro* (Atalay et al. 2002); however, the glycoproteins encoded by these genes play critical roles in the regulation of the host immune response *in vivo*. The RL11, RL12, and RL13 genes encode membrane glycoproteins, which bind the Fc region of IgG and prevent IgG-mediated clearance of the virus (Lilley et al. 2001, Cortese et al. 2012). Proteins UL7 and UL8 interact with a receptor on the surface of dendritic cells and activated neutrophils and impair their ability to secrete proinflammatory cytokines (Engel et al. 2011, Pérez-Carmona et al. 2018). Proteins UL10 and UL11 suppress proliferation and proinflammatory cytokine production in T cells (Gabaev et al. 2011, Bruno et al. 2016), while UL4 binds to tumour necrosis factor-related apoptosis inducing ligand (TRAIL) to prevent natural killer cell activation and TRAIL-mediated apoptosis (Vlachava et al. 2023). Thus, RL11 proteins perform a wide range of functions that are likely critical to evading and manipulating host immunity and promoting lifelong persistence of the virus.

To date, complete CMV genomes from 10 different species have been assembled. From these studies, we know that chimpanzee CMV, *Panine betaherpesvirus 2*, has 11 RL11 genes (Davison et al. 2003); African green monkey CMV, *Cercopithecine betaherpesvirus 5*, has 19 (Davison et al. 2013); rhesus macaque CMV, *Macacine betaherpesvirus 3*, and Japanese macaque CMV have 20 RL11 genes each (Davison et al. 2013, Taher et al. 2020). In contrast, CMVs of New World monkeys (NWMs) — owl monkey CMV, *Aotine betaherpesvirus 1*, and squirrel monkey CMV, *Saimiriine betaherpesvirus 4* — seem to lack RL11 genes (Davison et al. 2013). The same is true for the closely related betaherpesviruses of rodents (mouse, rat, and tupaia); their genomes do not carry RL11 genes (Davison et al. 2003). Genomes of several other Old World monkey (OWM) CMVs (*Papiine betaherpesvirus 4, Mandrilline betaherpesvirus 5*, and *Macacine betaherpesvirus 8*) are assembled (Marsh et al. 2011, Blewett et al. 2015), but the number of RL11 genes in these genomes is not clear because of the poor annotation of RL11 protein–coding genes, at least in part due to the extreme level of sequence divergence seen among this gene family.

It was reported that CR1 genes of human adenoviruses, encoded by the E3 genomic region, are potentially related to the RL11 gene family. CR1 genes also encode type 1 membrane glycoproteins with an SP, up to three Ig-like domains called CR1 domains, and a TMD (Davison et al. 2003). These genes, similar to members of the RL11 gene family, are dispensable for virus reproduction *in vitro* (Hitt et al. 1995), but not much is known about their role *in vivo* apart from the fact that they assist other genes located in the E3 region to perform immune evasion functions (Singh et al. 2013). Multiple sequence alignments (MSAs) show that CR1 and RL11D domain regions are formed around 15 loosely conserved residues, including one tryptophan and two cysteines present in the majority of proteins encoded by these genes (Davison et al. 2003). CR1 genes also show significant diversity between different genotypes, can be truncated, and/or lack one or more of their functional regions (Jacobs et al. 2004). However, the phylogenetic relationships between these genes and the RL11 gene family are currently unclear.

To our knowledge, the evolution of the RL11 gene family has not yet been investigated. Because of the low sequence conservation between members of this gene family and the relatively short length of the encoded proteins, elucidation of the phylogenetic relationships between these genes presents a challenging task. With recent advances in the field of protein structure prediction and the availability of new tools that help with the inference of structure-aware phylogenies, many evolutionary questions that could not be answered before with pure sequence–based approaches can be revisited. To collate a comprehensive dataset of RL11 genes, we performed a systematic *in silico* screening of genomes belonging to CMVs, related betaherpesviruses, mastadenoviruses, and their mammalian hosts. We conducted a traditional sequence–based and structure-guided phylogenetic analysis for the discovered members of the RL11 gene family to identify robust orthologs and guide our understanding of functional information within the gene family.

## Materials and methods

### In silico genome screening

A set of 34 betaherpesvirus genomes, 21 *Mastadenovirus* genomes, and 20 mammalian host genomes (see Supplementary Table S1) were analysed using database-integrated genome screening (DIGS) software v.2.0 (Blanco-Melo et al. 2024). DIGS is a genome screening pipeline based on the Basic Local Alignment Search Tool (BLAST) that allows users to perform iterative searches over genomes of interest using either protein or nucleotide probes and stores hits in a relational database. We conducted three screening iterations (Supplementary Fig. S1) on the same set of genomes, using empirically chosen thresholds based on multiple DIGS test runs. A tblastn bitscore threshold of 31 ensured a high diversity of nucleotide hits while minimizing false positives. A sequence length threshold of 85 bp captured core regions of the IgV-like domain or TMD, excluding shorter hits. A defragment range of 20 bp allowed the merging of two closely located hits into one while preventing the fusion of two nearby genes into a single hit. For the first search iteration, we used a probe set of 20 annotated RL11 protein sequences from *M. betaherpesvirus 3* strain 68-1 BAC (JQ795930.1) and 14 RL11 protein sequences from *H. betaherpesvirus 5* strain Merlin (AY446894.2). Although *H. betaherpesvirus 5* UL8 protein is known to be the result of splicing between the UL7 gene and a downstream located open reading frame (ORF) that encodes an alternative TMD, in this study, we used separate probes for the UL7 gene and the downstream ORF with alternative TMD, which we refer to as UL8 in the text. Coding sequences of newly discovered RL11 genes were translated *in silico* and added to the probe set used for subsequent search iterations. The RL11 proteins with a tblastn bitscore of >45 and a sequence length of >75 aa (Supplementary Table S2) discovered after the third iteration of screening were subjected to protein domain annotation, protein structure prediction, and phylogenetic analysis.

### Prediction of protein domains, functional regions, and glycosylation sites

We performed annotation of functional regions and protein domains for all *in silico* identified proteins using the web version of InterProScan 5 (Jones et al. 2014) in January 2024. TMDs were annotated based on TMHMM TMhelix coordinates from the InterProScan 5 results. For more accurate prediction of SPs, we submitted the same set of protein sequences to SignalP 6.0 (Teufel et al. 2022) in January 2024 with slow model mode and specifying Eukarya as an organism of choice. IgV-like domains could not be consistently predicted using InterProScan 5; therefore, for their annotation, we relied on MSAs and the predicted protein structures. We defined the IgV-like domain as a region encompassing cysteine 398 and valine 588 (alignment of RL11 proteins constructed with MAFFT v.7.475 Katoh and Standley 2013), which consists of nine beta strands and forms an IgV-like fold. To predict N- and O-linked glycosylation sites, we submitted the RL11 protein set to NetNGlyc 1.0 (Gupta and Brunak 2002) and NetOGlyc 4.0 (Steentoft et al. 2013) in January 2024.

### Protein structure prediction and structure-guided alignment

Protein structures for all 160 *in silico*-identified proteins were predicted using LocalColabFold v.1.5.3 (Mirdita et al. 2022) and ESMFold v.1.0.3 (Lin et al. 2023) software with default settings. ColabFold generally outperforms ESMFold in predicting high-quality protein structures. However, its performance depends on MSAs, leading to lower accuracy when publicly available databases lack sequences similar to the target protein. In contrast, ESMFold is a single-sequence structure predictor. It utilizes an ESM-2 transformer protein language model that does not require MSAs. This approach is advantageous for predicting proteins with low sequence similarity to publicly available sequences. In this study, we used a cut-off of an average predicted local distance difference test (pLDDT) score of 50, discarding structures of very low quality. Based on this criterion, 150 ESMFold and 130 ColabFold structures were considered suitable for the analysis. To maintain the diversity of protein structures in our analysis, we focused on the larger set of ESMFold predictions. Protein structures for representative members of each gene family and RL11 family clade with the highest pLDDT score were submitted to the PDBsum web server (Laskowski et al. 2018) in February 2024 to produce the topology maps of IgV-like domains.

Structure-guided pairwise sequence alignments of RL11 proteins were generated by Foldseek v.8.ef4e960 (Van Kempen et al. 2023) using easy-search with 3Di+AA option. It allowed us to produce pairwise local sequence alignments of protein regions based on their 3D structure for each pair of proteins. The structure with the highest average LDDT score across all pairwise structure alignments (*M. betaherpesvirus 1* RL11J) was used as a reference to create a joint structure–guided MSA.

### Phylogenetic analysis

MSAs were obtained for all RL11 proteins using MAFFT v.7.475 (Katoh and Standley 2013) with default auto settings. To preserve only reliable homologous regions, MAFFT and Foldseek alignments were processed with ClipKIT v.1.3.0 (Steenwyk et al. 2020) using default smart-gap option. The phylogenies were inferred from the ClipKIT-processed sequence alignments using IQ-TREE v.2.1.3 (Minh et al. 2020) with 100 transfer bootstrap replicates (Lemoine et al. 2018).

### Phylogenetic tree reconciliation

To determine when RL11 gene duplication events and losses took place during the evolution of CMVs, we used GeneRax v.2.0.4 (Morel et al. 2020) to perform reconciliation of the RL11 gene family phylogeny generated using the ClipKIT-processed MAFFT alignment and *Cytomegalovirus* phylogeny obtained from the ICTV web site (Lefkowitz et al. 2018). GeneRax allows users to produce species tree-aware phylogenies for gene families taking into account duplications, losses, and horizontal gene transfer events. We used GeneRax with rec-model UndatedDL option.

## Results

### Systematic *in silico* genome screening

We performed a systematic *in silico* screening of betaherpesvirus, *Mastadenovirus*, and mammalian genomes using a set of 34 annotated RL11 protein sequences from *H. betaherpesvirus 5* and *Mac. betaherpesvirus 3* as probes. The screening revealed 141 RL11 genes in the genomes of OWM CMVs and GA CMVs, 17 CR1 genes (CR1-β, CR1-γ, and CR1-δ) in the *Mastadenovirus* genomes and three genes from the EE50 gene family in the genomes of *Elephantid betaherpesvirus 1* and *E. betaherpesvirus 5* (Supplementary Fig. S2). The highest number of RL11 genes (20 genes) was found in the *Pap. betaherpesvirus 4* genome, while the lowest number (10 genes) came from the *Pan. betaherpesvirus 2* genome. The *Pan. betaherpesvirus 2* UL4 gene was not found in our screen. Also, we did not find any RL11-related genes in the genomes of the mammalian hosts. All the genes, apart from UL5, UL8 (ORF downstream of UL7, see Materials and methods), and RL11N, encoded proteins with at least one extracellular IgV–like domain. Some CR1 genes encoded proteins with two or even three consecutive Ig–like domains in their extracellular region (Supplementary Fig. S3).

### Phylogeny of the RL11, CR1, and EE50 gene families

**Figure 1.**
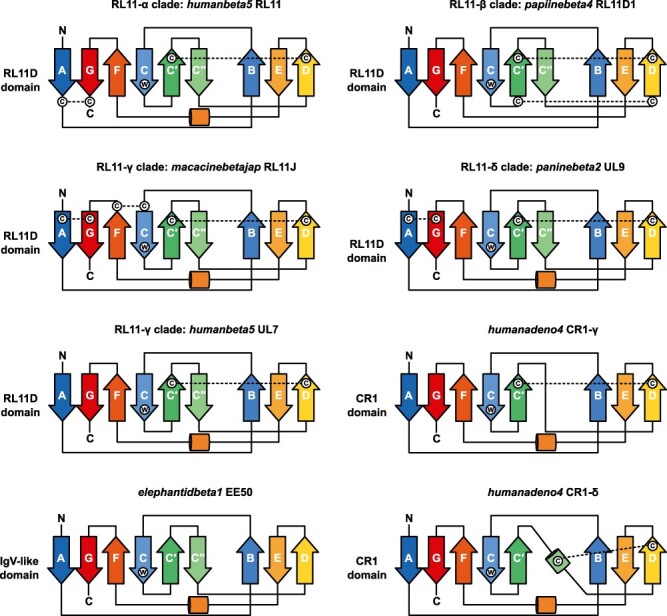
Molecular architecture of IgV-like domains from RL11, CR1 and EE50 protein families. Topology diagrams for members of the CR1, EE50 and different clades of the RL11 protein family with the highest pLDDT score of the protein model. RL11 proteins share a conserved disulphide bond between β-strands C’ and D. Arrows represent β-strands labelled according to the IgV domain convention (A, B, C, C’, C’’, D, E, F, G); cylinders represent α-helices; dashed lines represent predicted disulphide bonds; circled letters represent conserved cysteine (C) and tryptophan (W) residues; letters N and C represent N-terminal and C-terminal ends of IgV-like domains.

We performed a phylogenetic analysis to clarify the evolutionary relationships between the RL11, CR1, and EE50 gene families (Supplementary Fig. S4). According to the phylogeny, CR1 genes and EE50 genes formed a clade with UL7 and RL11P genes from the RL11 family, while a single CR1-δ gene from *Human adenovirus 4* was found within the RL11-γ clade of RL11 genes. However, because of the low bootstrap support of underlying nodes, the meaning of these phylogenetic relationships remains uncertain. To resolve this ambiguity, we predicted protein structures for all RL11, CR1, and EE50 proteins and produced topology maps for IgV-like domains from proteins of interest (Fig. 1). The topology diagrams show that IgV-like domains of analysed proteins usually consist of two β-sheets, with six β-strands in one sheet and three β-strands in the other with a conserved tryptophan residue in the β-strand C. Occasionally, the region corresponding to the sixth β-strand C″ can be absent completely (*H. adenovirus 4* CR1-δ) or be present but not form a β-strand (*H. adenovirus 4* CR1-γ).

All RL11 proteins together with some CR1-β and CR1-γ proteins also share a conserved disulphide bond between the β-strand C′ and the β-strand D that connects two β-sheets together. However, in contrast to RL11, CR1 proteins usually have more than one Ig-like domain. CR1-δ proteins have a disulphide bond in a similar place but between the β-strand D and a short α-helix downstream of the β-strands C′. CR1 domains of *H. adenovirus 4* CR1-δ and *Human adenovirus 3* CR1-δ proteins adopt analogous folds, so it is not clear why one protein was grouped together with other CR1-β and CR1-γ proteins, while the other was placed together with RL11 proteins (Supplementary Fig. S4). It is also surprising that UL7 and RL11P proteins form a clade with EE50 proteins. EE50 proteins of elephantid betaherpesviruses lack cysteine residues in the conserved positions of the IgV-like domain, a distinct mark of proteins encoded by CMV RL11 genes and *Mastadenovirus* CR1 genes. Therefore, the IgV-like domains of EE50 proteins lack disulphide bonds, while proteins UL7/RL11P share a conserved disulphide bond between the β-strand C′ and the β-strand D with other RL11 proteins (Fig. 1). These relationships are highly unstable in different phylogenetic reconstruction methods (sequence versus structure-aware, see Supplementary Fig. S5) and suggest that branches with significantly higher substitution rates are being pulled together in an artefact called long-branch attraction.

### Phylogeny of the RL11 gene family

Since even using protein structure information, we were unable to confidently establish the phylogenetic relationships between RL11, CR1, and EE50 gene families, we decided to focus our phylogenetic and synteny analyses on the RL11 genes of CMVs. We performed a sequence-based and structure-guided phylogenetic analysis on the RL11 proteins (Supplementary Fig. S5). Both phylogenies are broadly in agreement with the combined phylogeny of RL11, CR1, and EE50 proteins (Supplementary Fig. S4) and show that the RL11 gene family diversified into four major clades, which we called RL11-α, RL11-β, RL11-γ, and RL11-δ. However, due to the lack of high bootstrap support basally in the phylogeny, the relationships between these major clades are difficult to ascertain with confidence. Three of these clades (RL11-α, RL11-γ, and RL11-δ) have genes from both OWM CMVs and GA CMVs, while one clade (RL11-β) is unique to OWM CMVs. Both phylogenies consistently share the same sets of genes across the corresponding clades, apart from the unstable placement of the UL7 and RL11P genes, which belong to the RL11-γ clade in accordance with the structure-guided phylogeny or to the RL11-δ clade according to the sequence-based phylogeny.

According to the consensus between the two phylogenies, the RL11-α clade encompasses six genes (RL5A, RL6, RL11, RL12, RL13, and UL5) from the GA CMVs and three genes (RL11A, RL11G, and RL11T) from the OWM CMVs. The RL11-β clade is unique to the OWM CMVs and includes five genes: RL11B, RL11C, RL11D, RL11E, and RL11F. The RL11-γ clade is composed of two genes (UL4 and UL6) from GA CMVs and four genes (RL11H, RL11I, RL11J, and RL11R) from the OWM CMVs. The RL11-δ clade is the largest clade among the four, and it consists of five genes (UL1, UL8, UL9, UL10, and UL11) from the GA CMVs and eight genes (RL11K, RL11L, RL11M, RL11N, RL11O, RL11O2, RL11Q, and RL11S) from the OWM CMVs.

### Domain organization of RL11 proteins

**Figure 2.**
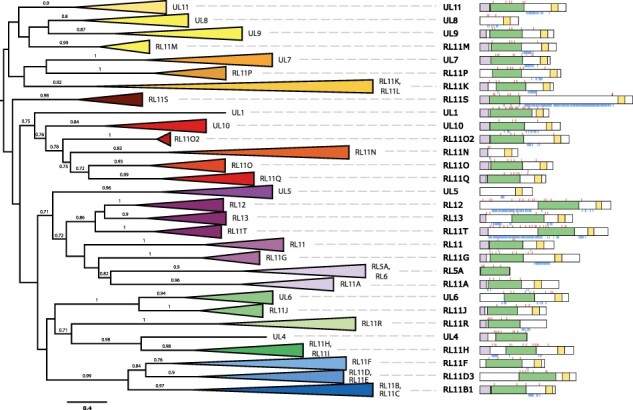
Evolution of CMV RL11 gene family. Midpoint rooted maximum likelihood phylogeny of amino acid sequences encoded by members of the CMV RL11 gene family (MAFFT alignment includes 141 sequences with 732 sites: 707 distinct patterns, 610 parsimony-informative, 92 singleton sites, 29 constant sites; substitution model: WAG+F+R5). Clades of orthologous genes are collapsed and coloured consistently with the synteny diagram in Fig. 3. Orthologs across OWM and GA CMVs have the same colour. Transfer bootstrap support values are shown only for nodes with support above 0.7. The scale bar represents number of amino acid substitutions per site. Schematic representation of RL11 protein structure for each clade is shown on the right (SP – lavender rectangle, RL11D domain – pistachio rectangle, TMD – pastel yellow rectangle, N-linked glycosylation sites – red marks on top, O-linked glycosylation sites – blue marks at the bottom). Schematic RL11 protein representations for GA CMVs come from *H. betaherpesvirus 5* (RL5A, RL6, RL11 – RL13, UL1 – UL11), protein representations for OWM CMVs come from *Mac. betaherpesvirus 3* (RL11O2) and *Pap. betaherpesvirus 4* (RL11A – RL11T). The domain coordinates for all proteins can be found in the Supplementary Table 3.

In addition to the phylogenetic analysis, we performed an *in silico* prediction of functional regions for proteins encoded by the CMV RL11 genes. Although most of the genes encode proteins with an SP, IgV-like domain (RL11D) and a TMD, many genes lack either one or two of these functional regions (Fig. 2). Since genes without some of these regions are related not to each other but to genes where all domains are present, we concluded that the loss of the functional regions occurred independently in different gene lineages. For instance, genes UL8 and RL11N encode only a TMD, but these genes are paralogous to UL9 and RL11O/RL11Q genes, respectively, encoding proteins with all three functional regions (SP, RL11D, and TMD). Another example involves genes RL5A and RL6, which encode only the RL11D region. These *H. betaherpesvirus 5*–specific genes are coorthologs (genes that emerged as a result of a lineage-specific duplication event and share orthology to one or more genes from another lineage) of the RL11A gene, which can be found in the OWM CMVs and encodes an SP, RL11D, and TMD. The same is true for the *H. betaherpesvirus 5*–specific gene UL4 that does not encode a TMD, but its coorthologs RL11H and RL11I do, and gene UL6 that lacks an SP, but its ortholog RL11J has an SP region.

Since RL11 genes encode transmembrane proteins, extracellular regions of these proteins are likely to be glycosylated. We conducted an *in silico* prediction of glycosylation patterns for all members of the RL11 protein family and noticed that N- and O-linked glycosylations are usually found in distinct regions of the protein. In general, N-linked glycosylation sites are enriched in the RL11D regions, while O-linked glycosylation sites are concentrated upstream and/or downstream of RL11D. In the context of glycosylation, three genes are of particular interest: RL12 and its ortholog RL11T have a long heavily O- and N-glycosylated region upstream of the RL11D, while RL11S specific to the OWM CMVs has a long heavily O-glycosylated region downstream of the RL11D.

### Duplications and losses in the RL11 gene family

**Figure 3.**
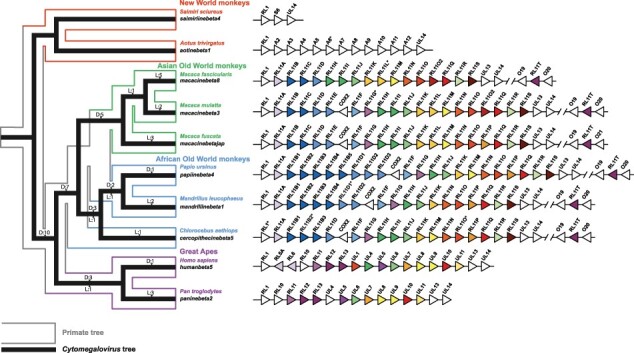
Gene synteny of RL11 family members in CMV genomes. Nested cladogram on the left reflects evolutionary relationships between CMVs (narrow black cladogram based on phylogeny from Lefkowitz et al. 2018) and their primate hosts (wide cladogram based on phylogeny from Kumar et al. 2022). The colour of the primate cladogram reflects if the host is a New World monkey (red branches), Asian Old World monkey (green branches), African Old World monkey (light blue branches), Great Ape (purple branches). Labels above and below branches indicate number of duplications (D) and losses (L) that occurred in the RL11 gene family before the lineage divergence. Gene synteny diagram on the right compares regions of CMV genomes where RL11 genes are located. All genes are represented by triangles to indicate direction of their transcription. Members of the RL11 gene family are coloured in accordance with their phylogenetic relationships (see Fig. 2), genes that do not belong to the RL11 family are kept white. Genes with deletions or internal stop codons are indicated with asterisks.

Analysis of the syntenic regions of the CMV genomes (Fig. 3) shows that although NWM CMVs (*S. betaherpesvirus 4* and *A. betaherpesvirus 1*) do not possess any members of the RL11 gene family in this region, they share genes flanking RL11 members (RL1 and UL14) with other CMVs. We also performed reconciliation of the RL11 gene phylogeny with the species phylogeny of CMVs (Supplementary Fig. S6) and added numbers of duplications and losses obtained from this analysis to the synteny diagram. The reconciliation analysis demonstrates that the RL11 gene family was shaped by several duplication events early in the evolution of OWM and GA CMVs, while losses of RL11 genes are generally more recent and usually lineage specific. For instance, 10 duplication events occurred before the divergence of OWM and GA CMVs and led to the formation of the four major clades of RL11 genes (RL11-α, RL11-β, RL11-γ, and RL11-δ).

Subsequent duplication events led to the expansion of different RL11 clades in different lineages of CMVs. The RL11-α clade expanded most significantly in the GA CMVs: *H. betaherpesvirus 5* genome contains six RL11-α genes, while genomes of other CMVs generally have three RL11-α genes. The RL11-β clade duplicated in the lineage of African OWM CMVs: the genome of *Pap. betaherpesvirus 4* carries nine RL11-β genes, while other CMVs usually have no more than five. RL11-γ and RL11-δ clades have the highest number of members in the Asian OWM CMVs: these CMVs acquired the RL11I gene through the duplication of the RL11H, the RL11L gene — via the RL11K duplication, and the RL11O2 gene after the duplication of either RL11O or RL11Q. However, genes related to the RL11M (RL11-δ clade) also expanded significantly in the GA CMVs leading to the emergence of UL8, UL9, and UL11 genes in *H. betaherpesvirus 5* and *Pan. betaherpesvirus 2*. Interestingly, *Mac. betaherpesvirus 8* does not have a genomic region encompassing three RL11-α and RL11-β genes (RL11E, RL11F, and RL11G), which are present in the *Mac. betaherpesvirus 3* and Japanese macaque CMV. The RL11O2 gene is unique to the *Mac. betaherpesvirus 3* and *Mac. betaherpesvirus 8*; it is found at the position of the RL11P gene, which is missing in these CMVs.

Multiple rounds of lineage-specific duplication events make the identification of orthologs between OWM CMVs and GA CMVs particularly challenging. Based on the branches with high bootstrap support (Fig. 2), we concluded that genes UL6 and RL11J have one-to-one orthologous relationship in all CMV genomes. This is similar to genes UL4 and RL11H, although in some CMVs the duplication of the RL11H gene led to the emergence of the RL11I gene, coortholog of UL4. Genes RL12 and RL13 found in the *H. betaherpesvirus 5* and *Pan. betaherpesvirus 2* are likely coorthologs of the RL11T gene specific to the OWM CMVs, while *H. betaherpesvirus 5*–specific genes RL5A and RL6 are coorthologs of the RL11A gene also found in the OWM CMVs. Orthologous relationships between other genes are less evident. UL7 may be an ortholog of the RL11P, genes UL8, UL9, and UL11 are likely related to the RL11M, and genes UL1 and UL10 have a close relationship to genes RL11O, RL11O2, RL11Q.

Another interesting observation is the distinct location of the RL11T gene in the genomes of the OWM CMVs. Unlike the rest of the RL11 genes, which are located in a row next to the left terminal repeat of the U_L_ region, RL11T is situated next to the right end of the U_L_ region close to the U_S_ region. The orientation of the RL11T gene also does not match the orientation of the other RL11 genes. *H. betaherpesvirus 5*–specific genes RL5A and RL6, although located at the same place as their ortholog RL11A in the OWM CMVs, have opposite orientations.

## Discussion

In this study, we performed a systematic *in silico* genome screening of CMVs, related betaherpesviruses, mastadenoviruses, and their mammalian hosts. We found RL11 genes in all analysed OWM and GA CMV genomes. The numbers of identified RL11 genes match previously reported data for *H. betaherpesvirus 5*, *C. betaherpesvirus 5*, *Mac. betaherpesvirus 3*, and Japanese macaque CMV (Davison et al. 2013, Taher et al. 2020). The gene annotated as UL4 in the *Pan. betaherpesvirus 2* genome was not detected in our analysis, indicating that this gene is a positional orthologue which is either unrelated to the RL11 family or pseudogenized beyond recognition. We also showed for the first time that *Pap. betaherpesvirus 4*, *Man. betaherpesvirus 5*, and *Mac. betaherpesvirus 8* have 22, 19, and 17 RL11 genes, respectively. We did not find RL11 genes in NWM CMV genomes nor in genomes of closely related betaherpesviruses (genera *Quwivirus, Muromegalovirus*, and *Roseolovirus*), confirming that these viruses are lacking RL11 genes (Davison et al. 2003).

It is worth mentioning that our genome screening approach was designed to find genes that encode proteins with noticeable sequence similarity to RL11 proteins. It was not designed to find all genes that encode proteins with structural similarity to RL11 proteins (e.g. possess IgV-like domain). Although we managed to find members of the EE50 gene family that encode proteins with IgV-like domains in *E. betaherpesvirus 1* and *E. betaherpesvirus 5* (genus *Proboscivirus*), the encoded proteins lack a disulphide bond between β-strands C′ and D, a characteristic mark of RL11 proteins, and were likely acquired by probosciviruses independently. We are aware that other viruses, not found in our study, may possess proteins with a similar protein structure. For example, NWM CMVs encode multiple proteins with IgV-like domains that share some structural similarity with RL11 proteins (Pérez-Carmona et al. 2015, Martínez-Vicente et al. 2019, 2020); however, the sequence similarity of these proteins was not sufficient to be identified in our screen. Thereby, we concluded that RL11 genes are specific to OWM and GA CMVs and they likely emerged after the divergence of NWM CMVs sometime between 42 and 29 million years ago (Kumar et al. 2022). Sequencing of CMV genomes infecting more basal primates such as loris, lemurs, and tarsiers will show if the RL11 gene family was a more ancient acquisition, which was lost in the NWM CMV lineage.

The high-sequence diversity and short-sequence length of the proteins encoded by RL11 genes make it challenging to establish phylogenetic relationships within the RL11 gene family with confidence; however, we managed to produce consistent phylogenies from a combination of sequence-based and structure-aware methods. We showed that the RL11 gene family forms four distinct clades (RL11-α, RL11-β, RL11-γ, and RL11-δ), with the RL11-β clade being unique to OWM CMVs. However, these phylogenies show low bootstrap support for basal nodes and, in some cases, suffer from long-branch attraction (Bergsten 2005) and, therefore, should be interpreted with caution. One possible remedy for addressing long-branch attraction involves increasing the taxonomic sampling, which helps to break long branches and improves the estimation of the substitution model parameter. This problem can be addressed by increased sampling of RL11 genes from CMVs that infect other GA and OWM hosts, such as orangutan, guenon, and colobus. Interestingly, several functional studies were conducted on the members of the RL11-α clade from *Mac. betaherpesvirus 3* (RL11A, RL11G, and RL11T), demonstrating the IgG Fc–binding activity characteristic for the members of the RL11-α clade from *H. betaherpesvirus 5* (RL11, RL12, and RL13) (Kolb et al. 2019, Taher et al. 2020, Otero et al. 2024). These findings clearly highlight the conservation of function of RL11-α genes across GA and OWM CMVs.

In our work, we defined the orthologous relationships between RL11 genes of OWM and GA CMVs. RL11 genes in these groups of CMVs are annotated in different ways. RL11 genes of OWM CMVs are named RL11A–RL11T that reflect their belonging to the RL11 gene family and the order in which these genes are located in the CMV genome. On the other hand, for historical reasons two different types of names are used to refer to RL11 genes in GA CMVs. Five gene names start with RL (RL5A, RL6, and RL11–RL13) and nine gene names start with UL (UL1, UL4, and UL5–UL11), where numbers are used to indicate the order of the genes in the genome and letters are used to refer to the region where they were initially found. UL stands for the U_L_ region and RL stands for the terminal repeat of the U_L_ (that was an artefact due to genome rearrangements in a passaged virus strain; in the wild-type CMVs, all RL11 genes are found in the U_L_). Our phylogenetic reconciliation analysis indicates that the RL11 gene family was shaped by a series of extensive duplication events early in the evolution of OWM and GA CMVs with the largest number of duplication events occurring basally following the divergence from NWM CMVs. Losses of various RL11 genes happened more recently and usually in a lineage-specific manner. Therefore, the RL11 gene family represents a complex case of evolutionary relationships between family members where most RL11 genes from GA CMVs do not have one-to-one orthologs with OWM CMVs, apart from several exceptions like RL11 and RL11G, UL4 and RL11H (some OWM CMVs have two coorthologs: RL11H and RL11I), UL6 and RL11J, and probably UL7 and RL11P.

In complicated cases like this, it is particularly important to carefully choose appropriate terms to reflect the relationships between specific family members. For instance, many functional studies of RL11 genes were performed without a defined phylogeny of this gene family and therefore suffered from incorrect assignment of orthologous/paralogous relationships between family members. Here, we would like to address some of them. First, our reconciliation analysis indicates that RL11G genes from OWM CMVs are orthologous to RL11 genes from GA CMVs and not to RL13 genes as was previously believed (Taher et al. 2020). This is interesting as previous studies showed that both RL13 and RL11G share a similar function of restricting the viral spread in fibroblasts (Schultz et al. 2020, Taher et al. 2020), suggesting that these genes might have independently acquired this function in GA (RL13) and OWM (RL11G) CMVs. It is also possible that other genes in the RL11-α clade share a similar ancestral function, but to our knowledge functional analysis of the latter has not yet been investigated.

Secondly, we showed that RL11T from OWM CMVs have two coorthologs in GA CMVs, RL13 and RL12, where RL12 is likely an isoortholog (coortholog that retains the structure and function of the ancestral gene after a duplication), while one-to-one orthologous relationship between RL12 and RL11T was previously assumed (Otero et al. 2024). Thirdly, the phylogenetic analysis shows that *H. betaherpesvirus 5*–specific genes RL5A and RL6 are coorthologs of the RL11A gene from OWM CMVs (but not necessarily isoorthologs), while before it was thought that the RL11A gene did not have an ortholog in GA CMVs (Kolb et al. 2019). Lastly, we would like to point out that according to our analysis, *H. betaherpesvirus 5*–specific gene UL1 is closely related to the UL10 gene where both genes likely emerged as the result of GA CMV–specific duplication event (Supplementary Fig. S6) and not as a duplication of RL11, RL12, or RL13 in the *H. betaherpesvirus 5* lineage as was previously speculated (Shikhagaie et al. 2012).

Although it was not the main focus of our study, we confirmed that CR1-β, CR1-γ, and CR1-δ genes in human adenoviruses and CR1-β genes in simian adenoviruses (genus *Mastadenovirus*) share noticeable sequence and structure similarity with RL11 genes. This finding is consistent with previously published data that human adenoviruses have genes potentially related to the RL11 gene family (Davison et al. 2003). We showed that although CR1 genes form a distinct clade on a phylogenetic tree, CR1-γ and CR1-β genes encode proteins with IgV-like domains that share the topology and conserved disulphide bond with RL11 proteins. CR1-δ genes, on the other hand, likely lost the region corresponding to the C″ β-strand because of a deletion, while the top part of the C′ β-strand carrying a conserved cysteine was adapted into an α-helix. Also, the majority of the CR1 proteins carry two Ig-like domains (IgV-like and IgC-like), while proteins with one, two, or three IgV-like domains are unique to human CR1-γ and human CR1-δ proteins. This could suggest that CR1-β genes with two Ig-like domains represent the ancestral form of CR1 proteins. We could not find CR1-α genes in our screen, suggesting that although these genes are related to CR1-β, CR1-γ, and CR1-δ, they share low-sequence similarity with RL11 genes and likely require a lowered *E*-value/bitscore threshold to be found with RL11 probes.

Although the relationship between CR1 and RL11 gene families remains unclear, it is striking that members of two distantly related families of DNA viruses that infect primates possess a set of proteins with a common 3D structure. We can see three possible scenarios of how this could have happened. The first scenario is that the founder gene of the CR1 and RL11 gene families is the same gene that was independently acquired by CMVs and mastadenoviruses from a primate host. In this case, we would expect that primates have a protein with the same IgV-like domain topology and a conserved disulphide bond in the same place. There are primate proteins that possess the same IgV-like domain topology, e.g. CD244/2B4 (Velikovsky et al. 2007) or CD226/DNAM-1 (Wang et al. 2019). Similar to the CR1-β proteins, both CD244 and CD226 have an IgV domain followed by an IgC domain in their extracellular regions, but these IgV domains have a disulphide bond in a different place. The extracellular region of the human CD229/SLAMF3/LY9, on the other hand, contains four Ig domains (IgV1, IgC1, IgV2, and IgC2) where IgV1 also shares the topology but not the disulphide bond with the RL11D and CR1 domains (Varadi et al. 2024). The second hypothesis is that the founder gene was acquired from a host only by one virus genus. In this virus genus, the gene underwent a rapid evolution and acquired a disulphide bond between β-strands C′ and D, which was later co-opted by another virus genus probably as a result of coinfection. The third scenario is that both CMVs and adenoviruses acquired two different genes with IgV-like fold from their hosts and adopted the same topology with a conserved disulphide bond through convergent evolution.

Each of these hypotheses relies on the virus acquisition of a host gene, and it is known that DNA viruses can co-opt mammalian genes via retrotransposition (Fixsen et al. 2022). Using our approach, which focused on understanding the evolution of the RL11 gene family in CMVs, we did not find any RL11-like matches in the mammalian hosts that satisfied our *E*-value/bitscore threshold. Future work using a protein-centred rather than a genome-centred screening approach might help to elucidate the mammalian origin of the RL11 gene family. However, we should point out that considering the high diversity of RL11 genes and the already limited bootstrap support of basal phylogenetic relationships, deciphering these deeper relationships with confidence will be challenging.

The extensive species–specific evolution of the RL11 gene family in primates during coevolution of their respective CMVs implies that these genes play key roles by interacting with components of the host immune system undergoing interdependent selection. Interestingly, not only is this gene family variable in sequence and arrangement between species, but the genes in the family are also the most highly variable between virus isolates within the same species. Many fall into 10 or more genotypes, suggesting that significant within-host evolution has also occurred since species divergence (Sijmons et al. 2015, Suárez et al. 2019). Identification of orthologs across different CMVs from this study may now facilitate dissection of functional homology and the underlying reason for this extensive lineage-specific gene diversification.

## Supplementary Material

veae066_Supp

## Data Availability

All data generated in this study including Python scripts, raw DIGS results, multiple sequence alignments, maximum likelihood phylogenetic trees, predicted protein structures, and predictions of functional regions are available at https://github.com/ulad-litvin/cmv_rl11_evolutionary_dynamics.
