# Supplementary material for: Evolution of the Cytomegalovirus RL11 gene family in Old World monkeys and Great Apes
Source: Virus Evol. 2024 Aug 24;10(1):veae066. doi: 10.1093/ve/veae066 (PMC11416908; doi:10.1093/ve/veae066)
Supplement: veae066_Supp [file veae066_supp.zip › supplementary_figures.docx]

**Evolution of the *Cytomegalovirus* RL11 Gene Family in Old World monkeys and Great Apes**

**Supplementary Data**

Ulad Litvin^1*^, Eddie C.Y. Wang^2^, Richard J. Stanton^2^, Ceri A. Fielding^2^, Joseph Hughes^1^

1 – MRC-University of Glasgow Centre for Virus Research, Glasgow G61 1QH, UK

2 – Infection and Immunity, Cardiff University School of Medicine, Cardiff CF14 4XN, UK

*Corresponding author: E-mail: [u.litvin.1@research.gla.ac.uk](mailto:u.litvin.1@research.gla.ac.uk)

**Supplementary Figures**


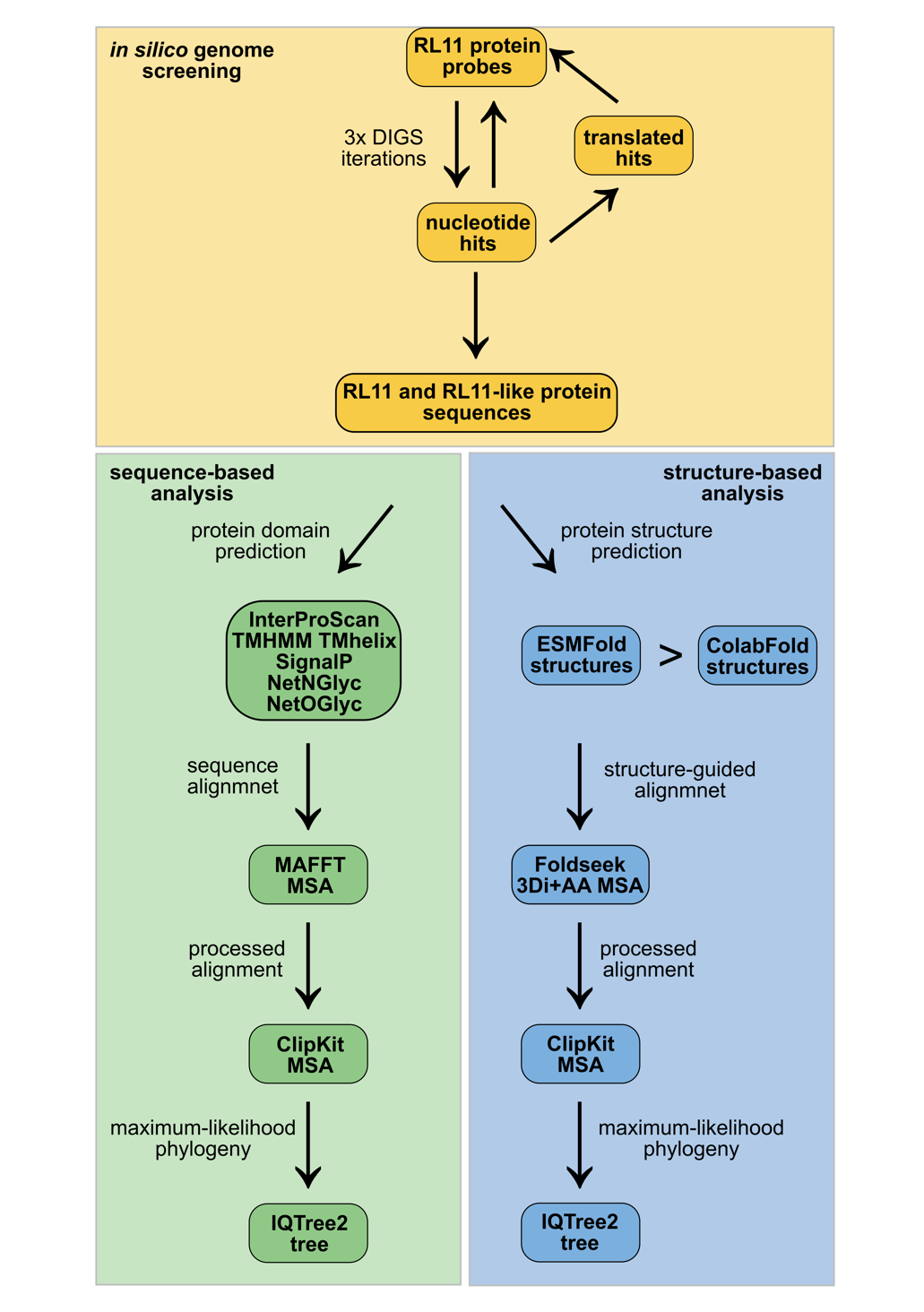


**Figure S1. Summary diagram of the computational analysis pipeline.** The performed analysis contained three main parts: *in silico* genome screening section (yellow boxes), sequence-based section (green boxes) and structure-based section (blue boxes). We started with a curated set of RL11 protein probes and used DIGS (Blanco-Melo et al. 2023) to screen viral and mammalian genomes. RL11 and RL11-like proteins found in the screen were subjected to protein domain prediction and protein structure prediction. After the domain prediction, protein sequences were aligned with MAFFT (Katoh & Standley 2013). The structural analysis of RL11 proteins was performed on structures predicted with ESMFold (Lin et al. 2023) because they demonstrated higher quality than ColabFold predictions (Mirdita et al. 2022). Structure-guided multiple sequence alignment (MSA) was produced using Foldseek 3Di+AA method (van Kempen et al. 2023). Both MSAs were processed with ClipKit (Steenwyk et al. 2020). Phylogenetic inference was performed using IQTree2 (Minh et al. 2020).


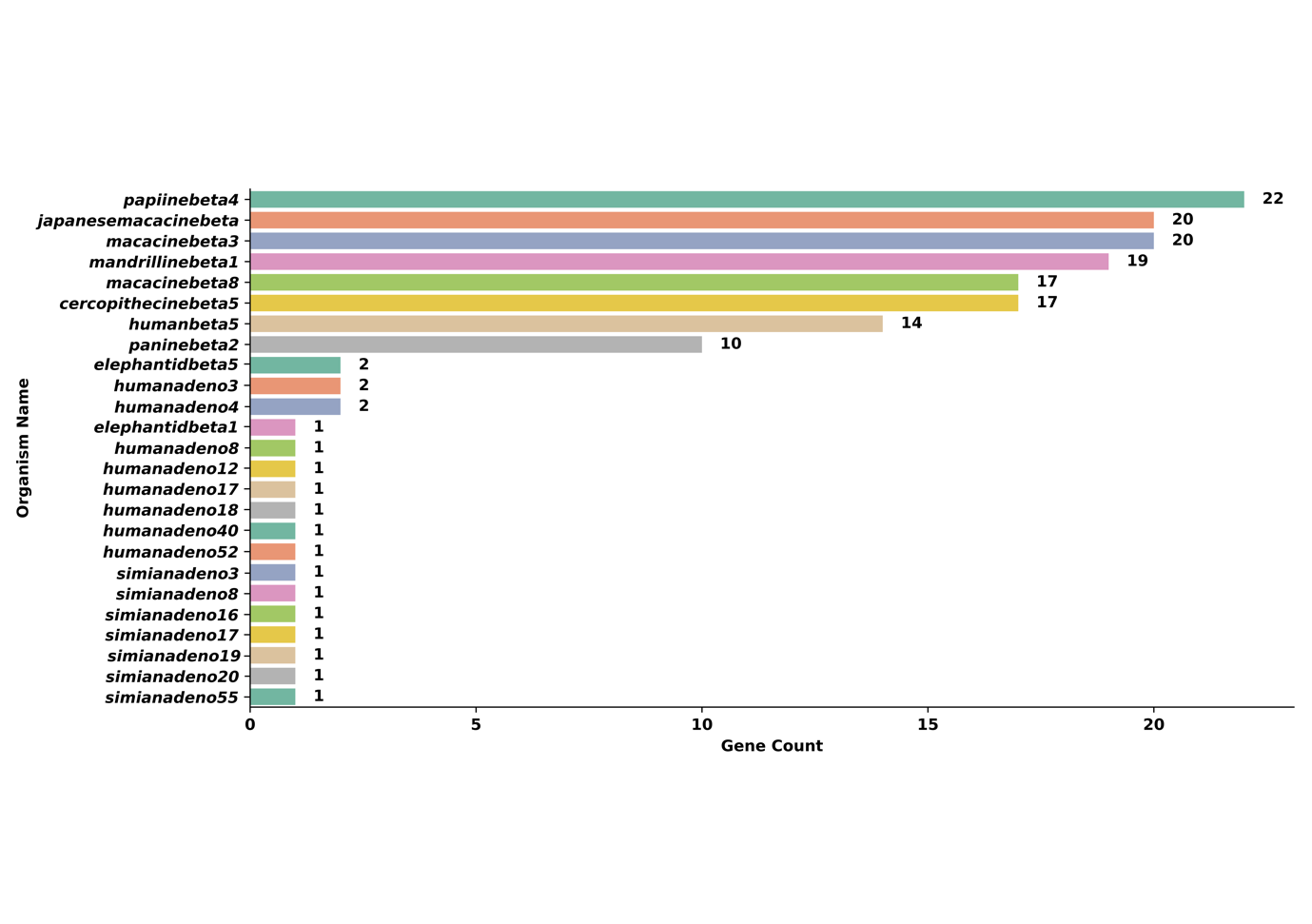


**Figure S2. Distribution of RL11-like genes found with *in silico* genome screening.** Each bar shows number of RL11, CR1, EE50 genes identified by *in silico* genome screening for each *Cytomegalovirus*, *Mastadenovirus*, *Proboscivirus* species. In total 141 RL11, seventeen CR1 and three EE50 genes were found. The number of RL11 genes per genome varied significantly from ten genes in *paninebeta2* genome to twenty-two genes in *papiinebeta4* genome.


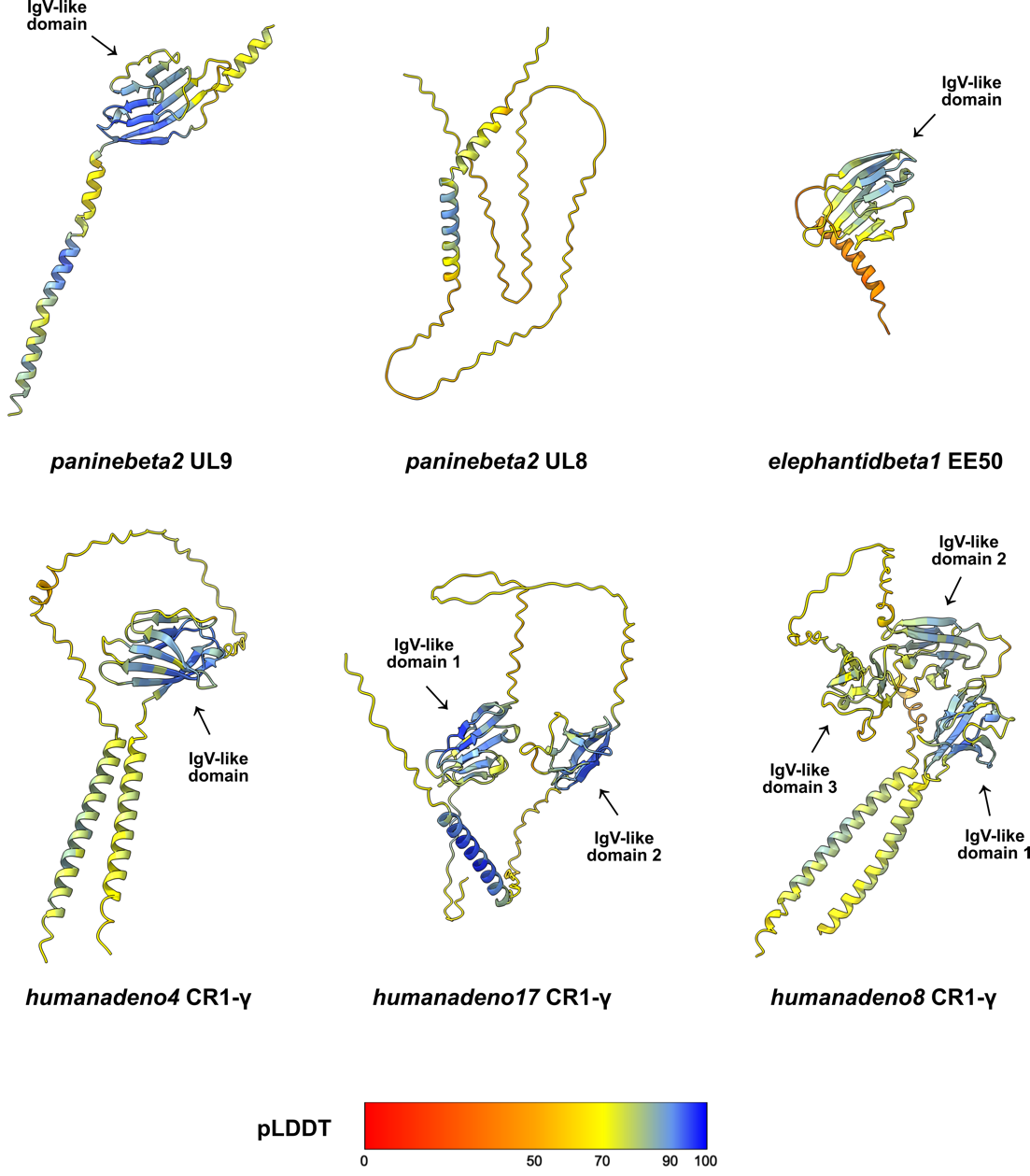


**Figure S3. Number of IgV-like domains in CR1, EE50 and RL11 proteins.** Ribbon diagrams of CR1, EE50 and RL11 protein structures predicted with ESMFold (Lin et al. 2023). IgV-like domains are indicated by arrows. Number of IgV-like domains vary between proteins: all EE50 proteins have one IgV-like domain; all RL11 proteins apart from UL5, UL8 (ORF downstream of UL7) and RL11N have one IgV-like domain; number of IgV-like domains in CR1 proteins varies from one (eg. in *humanadeno4* CR1-γ) to three (eg. in *humanadeno8* CR1-γ). Models are coloured by pLDDT confidence score: blue (very high confidence), cyan (high confidence), yellow (low confidence) and red (very low confidence). All predicted structures are available on GitHub: https://github.com/ulad-litvin/cmv_rl11_evolutionary_dynamics/tree/main/esmfold


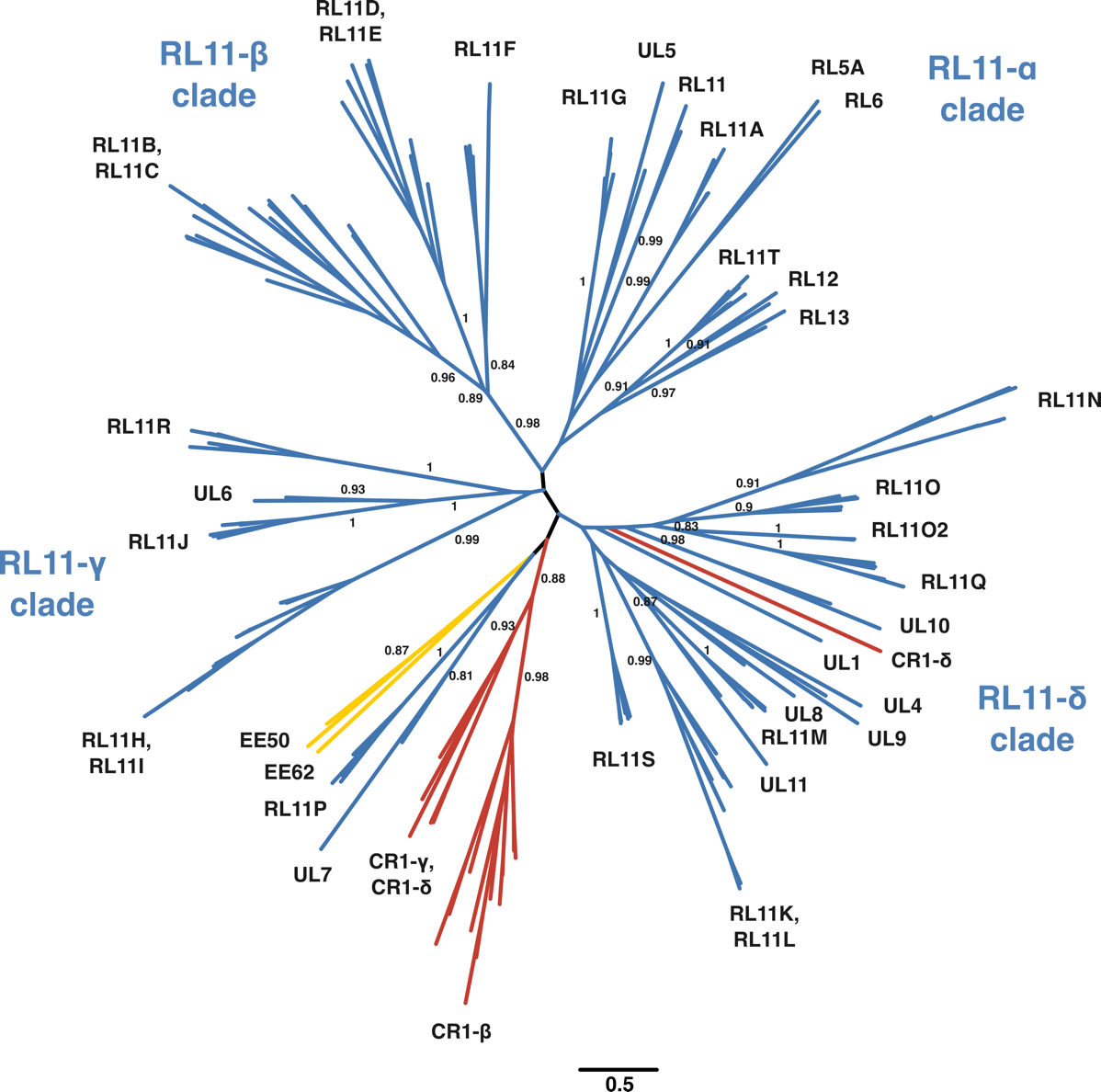


**Figure S4. Unrooted phylogeny of CR1, EE50 and RL11 gene families.** Unrooted maximum likelihood phylogeny of amino acid sequences encoded by CR1, EE50, RL11 genes identified in the DIGS screen and inferred from MAFFT alignment (alignment includes 160 sequences with 779 columns, 764 distinct patterns, 677 parsimony-informative, 76 singleton sites, 26 constant sites; IQTREE optimal log-likelihood of the consensus tree: -69675.328; substitution model: WAG+F+R5). Branches are coloured to reflect if a gene belongs to the CR1 (red branches), EE50 (yellow branches) or RL11 (blue branches) gene family. Transfer bootstrap support values are shown only for nodes with support above 0.8 (values for nodes within clades of orthologous genes are not shown). Scale bar represents number of substitutions per site.


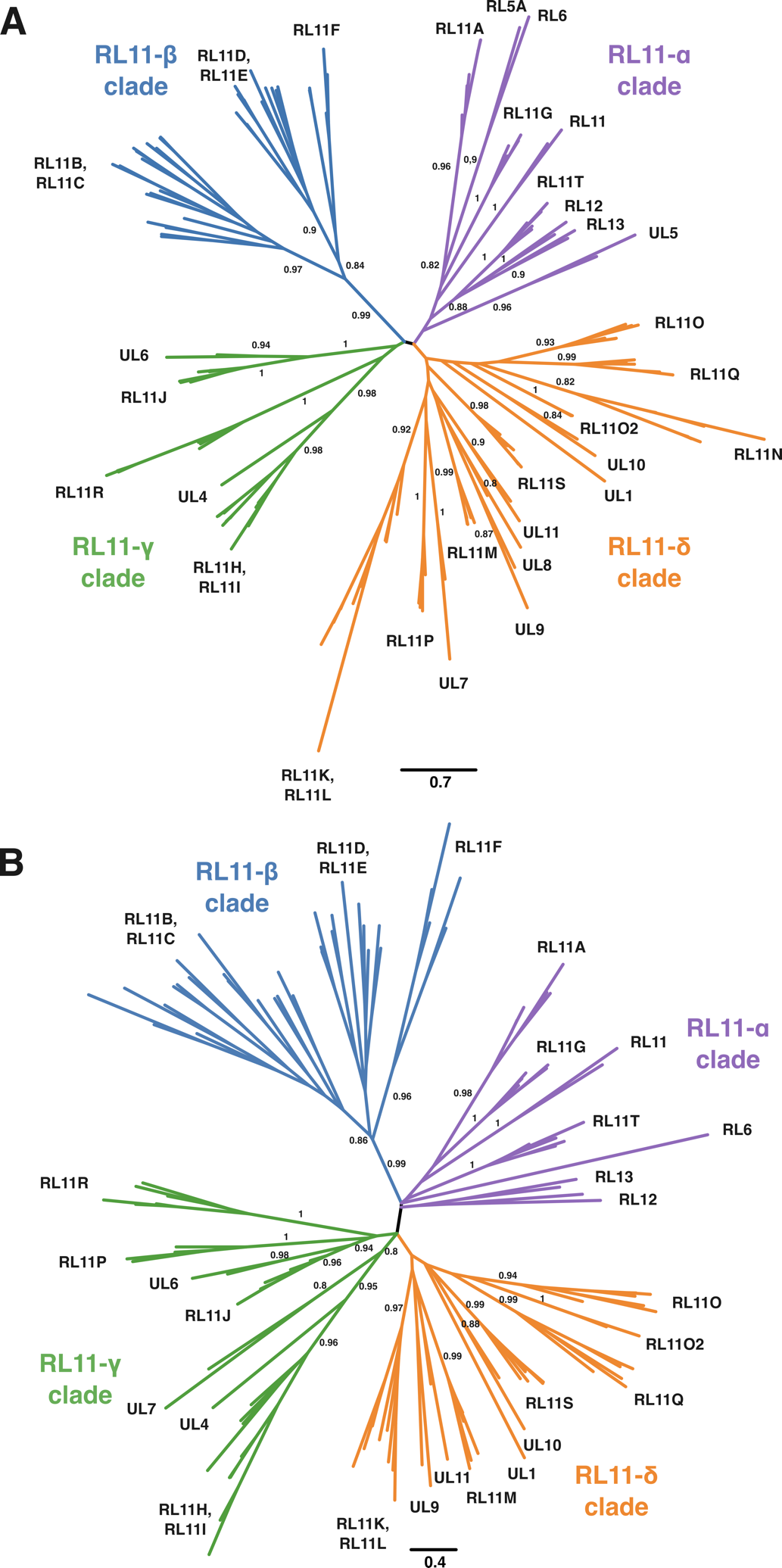


**Figure S5. Unrooted phylogenies of *Cytomegalovirus* RL11 gene family.** **(A)** Unrooted maximum likelihood phylogeny of all amino acid sequences encoded by *Cytomegalovirus* RL11 genes inferred from MAFFT alignment (alignment includes 141 sequences with 732 sites: 707 distinct patterns, 610 parsimony-informative, 92 singleton sites, 29 constant sites; IQTREE optimal log-likelihood: -59286.757; substitution model: WAG+F+R5). **(B)** Unrooted maximum likelihood phylogeny of amino acid sequences with IgV-like domain encoded by *Cytomegalovirus* RL11 genes inferred from Foldseek alignment (alignment includes 129 sequences with 181 sites: 181 distinct patterns, 179 parsimony-informative, 2 singleton sites, 0 constant sites; IQTREE optimal log-likelihood: -42848.800; substitution model: VT+F+R6). RL5A protein has been excluded from the alignment because of the low pLDDT score of the model (below 50). Branches are coloured to reflect divergence of RL11 genes into four major clades (RL11-α, RL11-β, RL11-γ, and RL11-δ) based on the overall topology of the tree. Transfer bootstrap support values are shown only for nodes with support above 0.8 (values for nodes within clades of orthologous genes are not shown). Scale bar represents number of substitutions per site.


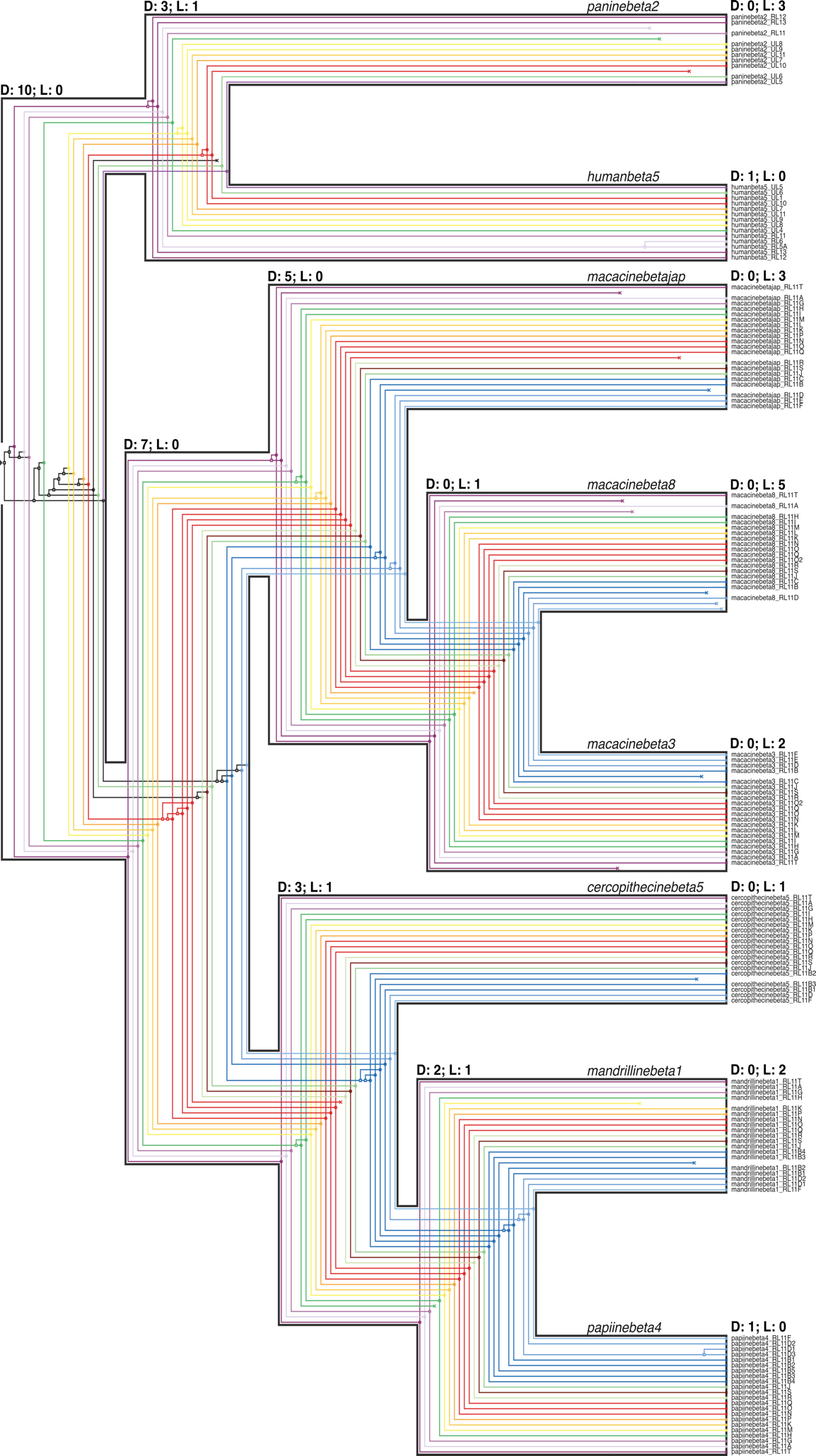


**Figure S6. *Cytomegalovirus* species-tree aware phylogeny of the RL11 gene family**. Unrooted maximum likelihood phylogeny of all amino acid sequences encoded by *Cytomegalovirus* RL11 genes inferred from MAFFT alignment has been reconciled with *Cytomegalovirus* species tree (Lefkowitz et al. 2018) using GeneRax (DT rates: D=0.129934, L= 0.0876392; reconciliation likelihood: -173.64; phylogenetic likelihood: -59997.4; joint likelihood: -60171). Black cladogram represents *Cytomegalovirus* phylogeny. RL11 gene family phylogeny is coloured consistently with Figure 2 (empty square nodes represent gene duplication events, filled circle nodes – speciation events, crosses – gene loss events). Number of gene duplication events (D) and gene loss events (L) are summarised on top of each node.
